# Nutritional and physicochemical changes in two varieties of fonio (*Digitaria exilis* and *Digitaria iburua*) during germination

**DOI:** 10.1016/j.heliyon.2023.e17452

**Published:** 2023-06-19

**Authors:** Stella Oyom Bassey, Chiemela Enyinnaya Chinma, Vanessa Chinelo Ezeocha, Olajide Emmanuel Adedeji, Olusola Samuel Jolayemi, Uzoamaka Christa Alozie-Uwa, Irene Eneyi Adie, Salvation Isang Ofem, Janet Adeyinka Adebo, Oluwafemi Ayodeji Adebo

**Affiliations:** a Department of Human Nutrition and Dietetics, Faculty of Basic Medical Sciences, University of Calabar, Nigeria; b Department of Food Science and Technology, Federal University of Technology Minna, Nigeria; c Department of Biotechnology and Food Technology, University of Johannesburg, P. O. Box 17011, Doornfontein Campus, Gauteng, South Africa; d Department of Food Science and Technology, Michael Okpara University of Agriculture Umudike, Nigeria; e Department of Food Science and Technology, Federal University Wukari, Wukari, Nigeria; f Department of Food Science and Technology, Federal University of Technology Akure, Nigeria; g Food Evolution Research Laboratory, Bunting Campus, School of Hospitality and Tourism, College of Business and Economics, University of Johannesburg, P.O. Box 17011, Johannesburg 2028, South Africa; h Food Innovation Research Group, Department of Biotechnology and Food Technology, University of Johannesburg, P. O. Box 17011, Doornfontein Campus, Gauteng, South Africa

**Keywords:** Fonio, *Digitaria exilis*, *Digitaria iburua,* germination, Nutritional composition, Techno-functional properties

## Abstract

Germination is a cheap and effective bioprocessing technique used for improvement of the nutritional, physicochemical and health-promoting properties of seeds. The benefits of germination on two fonio varieties (*Digitaria exilis* and *Digitaria iburua*) have not been studied. This study investigated the nutritional and physicochemical changes in two varieties of fonio germinated for 24, 48 and 72 h at 28 °C. The antioxidant, protein and starch digestibility, functional, pasting, and thermal properties were also determined. Germination over time (24, 48, 72 h) significantly (p ≤ 0.05) increased the protein, ash, total dietary fiber, majority of the amino acids, minerals, protein digestibility, resistant starch, total phenolics and antioxidant activities while phytic acid, tannin, saponin and digestible starch contents decreased in both varieties. Germination significantly increased water and oil absorption capacity, and slightly modified pasting and thermal characteristics while bulk density decreased in both varieties. The principal component analysis revealed germination time to be the key determinant in the physicochemical, nutritional, and techno-functional characteristics of fonio rather than variety, with raw and 24 h germinated grains having similar attributes. The study established that germination improved the nutritional, antioxidant, and techno-functional properties of *Digitaria exilis* and *Digitaria iburua*, which can serve as novel food ingredients for product development.

## 1 Introduction

Recently, there has been an increasing trend towards the modification of cereal grains for improved nutrition, as well as enhancement of the techno- and bio-functionality of the resulting flours and products [1]. Germination is a bioprocessing technique which involves conditioning cereals at high humidity (>70%) and mild temperature (25−40 °C) to stimulate hydrolytic enzymes such as amylases, glucanases and proteases to modify the endosperm [2]. This is accompanied by the breakdown of macromolecules and the release of key metabolites such as amino acids and bioactive compounds, improving nutrient density, bioavailability, and bioactivity [3]. In addition, structural modification takes place during cereal grain germination, enhancing the physicochemical and cooking qualities of products [4]. Relative to other cereal grain-modifying techniques, such as fermentation and extrusion, germination processes are cheaper, more easily adaptable, and generate little effluent [5]. Several studies have demonstrated improved nutritional and functional properties of common cereal grains such as wheat, brown rice, oat, and sorghum following germination [3,5,6]. However, more studies are required to explore novel and underutilized cereal grains.

White fonio *(Digitaria exilis)* and brown fonio (*Digitaria iburua)*, the two commonly cultivated fonio varieties in West Africa [7], are attracting research attention due to their low gluten content and glycemic index and rich phytochemicals, encouraging their use in functional foods formulation [8]. They are also a good source of amino acids (methionine and cysteine) and minerals [9], although poor nutrient bioavailability due to antinutritional factors such as phytates, has been reported [10]. Therefore, modification of the grains for improved nutritional quality is imperative. Some studies have reported differences in the physicochemical properties of the two fonio varieties [9,10]. Studies exploring the grains in product development are well documented [7,11,12], however, information relating to the influence of germination on the nutritional composition and physicochemical changes of the two fonio varieties is sparse.

This study aimed to evaluate the influence of germination time (0−72 h) on the nutritional and physicochemical properties of two varieties of fonio, *Digitaria exilis* and *Digitaria iburua*. During germination, the grains were profiled for proximate starch, dietary fiber, amino acids, minerals, antinutrients, and antioxidants. *In vitro* starch digestibility, techno-functional, pasting, and thermal properties were also evaluated.

## 2 Materials and methods

### 2.1 Germination of the grains and production of flours

The grains were procured from Minna, Nigeria, and germinated according to a procedure by Cornejo et al. [2]. 250 g of the grains were initially washed with 0.07 g/L hypochlorite solution for 30 min, for sterilization purposes, and then drained. Thereafter, the grains were soaked in distilled water (1 L) for 7 h at 28 ± 1 °C. The hydrated grains were subsequently germinated in a chamber at 28 ± 1 °C and 92% relative humidity under darkness for 24, 48 and 72 h. Germination of the grains was carried out in triplicate and germinated grains were then sorted and oven dried (Gallenkamp 300 series, Widnes, Cheshire, UK) at 40 °C for 24 h. Germinated and raw (control) grains were milled, sieved (100 μm screen mesh) and kept in labelled airtight containers at 4 °C until further analysis.

### 2.2 Macronutrient and dietary fiber analyses

The flour samples were analyzed using standard methods [13] for moisture (method no. 925.09B), ash (method no. 923.03), fat (method no. 920.39C,), protein (method no. 992.23), soluble dietary fiber (SDF) and insoluble dietary fiber (IDF) (method no. 991.43) and total dietary fiber (calculated as sum of IDF and SDF) [13]. The amylose content was analyzed using the iodine method of Williams et al. [14], by first dispersing the samples in 0.5 N potassium hydroxide and then adding 0.1 N hydrochloric acid. An iodine reagent was then added to the mixture and the absorbance (625 nm) of the complex was measured in a spectrophotometer. Total sugar was assayed following the phenol-sulfuric method of Sadasivam and Manickam [15]. Briefly, 2 g of sample was extracted with 80% ethanol, then centrifuged, and the supernatant was collected. Thereafter, an aliquot (0.1 and 0.2 mL) was taken in separate test tubes and made up to volume (10 mL) with distilled water. 1 mL of 5% phenol was added to each test tube, followed by the addition of sulfuric acid (5 mL) to each test tube after 10 min. The absorbance (490 nm) was measured in a spectrophotometer.

### 2.3 Mineral analysis

Mineral constituents [zinc (Zn), calcium (Ca), magnesium (Mg), potassium (K) and iron (Fe were investigated using AAS (atomic absorption spectrophotometer, 2380, PerkinElmer, Massachusetts, USA) using the No. 985.35 protocol of AOAC [13]. The content of P (phosphorus) was assayed using the flame photometric method (No. 984.27) [13]. The results were expressed in mg/100 g.

### 2.4 Antinutritional factors

#### 2.4.1 Phytic acid

The phytic acid (PA) in the sample (extracted by mixing 1 g of flour with 20 mL HCl) was measured in a UV/Vis spectrophotometer (Genesys G10S, Thermo Fisher Scientific, Waltham, USA) at an absorbance of 500 nm, following the procedure described by Chinma et al. [16]. The phytate value was measured from the phytic standard curve (y = 0.513x + 0.0438, R^2^ = 0.9978) and the results were reported in mg/100 g.

#### 2.4.2 Tannin

Tannin was extracted by mixing 0.5 g of flour with methanol (5 mL of l%, v/v), and was measured at an absorbance of 760 nm in a UV–Visible spectrophotometer (G10S, Thermo Fisher Scientific, Waltham, USA) [17]. Tannin content was measured from a standard curve of catechin (y = 0.0093x + 0.0541, R^2^ = 0.99504). The results were presented in CE/100 g.

#### 2.4.3 Total saponin

The total saponin in the sample was assayed using a method reported by Lai et al. [18]. A 0.5 g sample was mixed with petroleum ether (10 mL), followed by shaking at room temperature (28 ± 2 °C) for 4 h. Thereafter, 20 mg of sample was extracted with 80% (v/v) aqueous methanol with shaking for 4 h, and the extract was kept at 4 °C in the dark prior to analysis. Absorbance was measured using a UV–Visible spectrophotometer (G10S, Thermo Fisher Scientific, Waltham, USA) at 760 nm. The results were expressed as mg/100 g sample.

### 2.5 Amino acid (AA) analysis

A standard method by Bidlingmeyer et al. [19] was used. Briefly, this involved protein hydrolysis, using 6 M hydrochloric acid at 110 °C for 24 h, precolumn derivatization and analysis by reverse phase high-performance liquid chromatograph coupled with a photodiode array detector at 254 nm [20]. The results were expressed in g/100 g.

### 2.6 *In* vitro protein digestibility (IVPD)

A standard method detailed by Chinma et al. [16] was used for IVPD determination. A 200 mg sample was placed in an Erlenmeyer flask (100 mL) containing 35 mL of 0.1 mol/L sodium citrate tribasic dihydrate at pH 2.0, and 1.5 g/L pepsin. The mixture was incubated at 37 °C for 2 h, centrifuged (15 min, 10,000×*g*), and the supernatant was decanted. Thereafter, the residue was washed, dried, and assayed for nitrogen value [13]. Percentage IVPD of the samples was calculated using Eq. 1

(1) $$IVPD=\frac{\left(total\;nitrogen-residue\;nitrogen\right)\times 100\%}{total\;nitrogen}$$

### 2.7 *In vitro* starch digestibility

Starch digestibility was carried out following the method detailed by Englyst et al. [21] under controlled enzymatic hydrolysis, followed by colorimetric measurement of the glucose released (using glucose oxidase-peroxidase, GOPOD kit, KGLOX, Megazyme, Wicklow, Ireland). The rapidly digested starch (RDS), slowly digested starch (SDS), and resistant starch (RS) were calculated as reported by Englyst et al. [21].

### 2.8 Total phenol and antioxidant activities

The extraction of sample (0.2 g) was carried out in 4 mL of 80% aqueous methanol [16]. The methanolic extracts of the samples were thereafter used for the profiling of total phenolic content (TPC) and antioxidant activities (1,1-diphenyl-2-picryl-hydrazil radical scavenging activity, DPPH; oxygen radical absorbance capacity, ORAC; and ferric reducing antioxidant power, FRAP). The TPC was assayed in a UV–Visible spectrophotometer as described by Singleton and Rossi [22]. The results were reported in mg/GAE/100g calculated from a calibration curve (y = 1.1319x + 0.0563, R^2^ = 0.9955). The DPPH radical scavenging activity analysis was performed at an absorbance of 516 nm in a UV–Visible spectrophotometer following an official procedure detailed by Brand-Williams et al. [23] while the DPPH value was measured using Trolox as a standard, and results were reported as μmol TE/100g, calculated from a calibration curve (y = −0.4581x + 0.7284, R^2^ = 0.9967). The FRAP was measured as detailed by Oyaizu et al. [24]. The results were reported in μmol TE/100 g, calculated from a calibration curve, y = 1.4290x + 0.6341 while R^2^ = 0.9932. The ORAC was assayed following the method of Ou et al. [25]. The results were reported as μmol Trolox equivalent/100 g obtained from a calibration curve (y = 1.1945x + 0.6520, R^2^ = 0.9974).

### 2.9 Functional characteristics

The bulk density, expressed as weight of sample per volume of sample, was determined as detailed by Azeez et al. [20], while oil (OAC) and water absorption capacity (WAC), expressed as weight of water/oil absorbed per unit weight of sample, was determined following a standard procedure of AACC 56–20, [26]. The water solubility index (WSI) was determined as reported by Cornejo and Rosell [27].

### 2.10 Pasting analysis

Pasting characteristics of the flour were profiled with a rapid visco-analyzer (RVA, Newport Scientific Pty Ltd., New South Wales 2102, Australia). Distilled water (25 mL) was added to the sample (2.5 g) in the RVA canister, and the resulting slurry was mixed and then placed in the RVA. The RVA temperature program was set as follows: initially kept at 50 °C for 1 min, then heated to 95°at 12.2 °C/min and held at 95 °C for 2.5 min, then later cooled to 50 °C for 2 min at the rate of 11.8 °C/min [16]. During the cycle, the paste viscosities (peak, breakdown, trough, setback, peak and final viscosity), pasting temperature and peak time were recorded [20].

### 2.11 Thermal profiling

Thermal profiling of the flour was done in a differential scanning calorimeter, DSC model 204, Nietzsche, Germany, using 5 mg of sample mixed with distilled water at a ratio of 1:3 (w/v), and sealed in a hermetic pan as detailed by Azeez et al. [20]. Thereafter, the sample was heated from 25 °C to 120 °C at a rate of 10 °C/min. The onset, peak and conclusion temperatures, and gelatinization enthalpy were recorded.

### 2.12 Data analysis

Analyses were conducted in triplicate and reported on a dry weight basis. Data obtained were subjected to analysis of variance using SPSS version 20, (IBM, Armonk, USA). Differences among the means of the parameters (of both brown and white fonio varieties) were separated using Tukey's test at 5% probability. For multivariate data analysis, principal component analysis (PCA) was used to capture the unsupervised empirical pattern distribution of the fonio flour samples based on changes in their physicochemical, functional, and pasting properties. As an exploratory statistical tool, PCA was used to generate a simplified one-dimensional representation (score and loading plots) of data as a function of essential latent variables known as principal components (PCs) or eigenvalues. In addition, the Partial Least Squares−Variable Importance in Projection (PLS-VIP) technique was utilized to prioritize measured variables in order of their relevance on a pre-selected index [28] (Mukherjee et al., 2015). The selected indices (Y variable) chosen (supervised model) were *in vitro* starch and protein digestibility, owing to their nutritional importance. The importance of the variables was established by correlating their variations (weights) to the Y variable. A VIP weight is considered significant if it is greater than one (1) [29]. The magnitude and direction of a VIP coefficient determines the influence of the independent variable (X) and the dependent variable (Y). The higher the coefficient, the higher the influence will be; an upward pointing bar has a positive influence, while a downward pointing one has a reverse relationship. The model’s performance indexes were R^2^ (coefficient of prediction), RMSEE (root mean square error of estimation) and RMSE_CV_ (root mean square error of cross-validation). The PCA and PLS-VIP models were analyzed using the SIMCA 14.1 (Umetrics, Umea, Sweden) statistical package.

## 3 Results and discussion

### 3.1 Proximate composition

The proximate constituents of the fonio grains were influenced by germination time and grain variety (Table 1). The moisture content of the samples was within acceptable limits for storability (<10.50 g/100g) [9]. Compared to the raw fonio, the available protein content increased as germination progressed, with up to 63.20% and 65.32% increase in the brown and white varieties, respectively, after 72 h germination. This was likely due to the release of protein in the seed structure during the breakdown of the starch granule by α-amylase during germination [20] or possibly due to protein synthesis during germination [16]. Enzymes are generated during germination and these enzymes act on available nutrients (starch, carbohydrates, fats and proteins) leading to a change in composition of the other constituents, as well as the possible presence of newly formed proteins [30]. This concurs with the findings of Jimenez et al. [4] on quinoa and amaranth grains. The fat content of the raw/germinated brown (2.50−4.10 g/100 g) and white (2.31−4.26 g/100 g) fonio varieties (Table 1) is similar to the 1.1%–4.7% and 1.3%–4.3% reported for white and black varieties of fonio respectively in other studies [9]. Fat content reduced as germination progressed in both varieties as a result of the increased activities of lipolytic enzymes, which break down fat into glycerol and fatty acids [4] and is used as an energy source during germination. A similar observation was made by Azeez et al. [20] who noted a 21.4% reduction in fat content in brown finger millet after 48 h of germination. The ash value, an indication of mineral content of a food material, was significantly higher in the raw brown variety of fonio than the raw white variety, suggesting that the mineral content of the raw brown variety may be higher than that of the raw white variety. The ash content of the samples increased as germination progressed, by 43.88% and 43.97% for the brown and white varieties, respectively, which was attributed to the increased activity of enzymes (such as phytase) during sprouting, leading to the hydrolysis of phytic acid and liberating the ash-related constituents, thus increasing the ash contents [31]. Soluble, insoluble, and total dietary fiber content increased (p ≤ 0.05) with an increase in germination time in both varieties. The increase in soluble fibre may be explained by the rise of cellulosic glucose due to the metabolic reactions undergone by the seeds during germination, while the increase in insoluble and total dietary fiber may be due to the formation of cell walls as the radicle developed [20].

**Table 1:** Chemical composition (g/100 g) properties of two varieties of native and germinated fonio flour.

Samples	Brown variety				White variety			
	Raw	24 h	48 h	72 h	Raw	24 h	48 h	72 h
Moisture	10.33 ± 0.00^a^	10.12 ± 0.03^b^	10.17 ± 0.01^b^	10.19 ± 0.01^b^	10.38 ± 0.02^a^	10.20 ± 0.05^b^	10.16 ± 0.01^b^	10.24 ± 0.03^b^
Protein	8.56 ± 0.01^e^	10.79 ± 0.02^c^	12.23 ± 0.05^b^	13.97 ± 0.01^a^	8.39 ± 0.01^e^	9.47 ± 0.01^d^	12.10 ± 0.03^b^	13.87 ± 0.02^a^
Fat	4.10 ± 0.02^a^	3.54 ± 0.03^b^	2.81 ± 0.02^c^	2.50 ± 0.00^d^	4.26 ± 0.03^a^	3.80 ± 0.02^b^	3.04 ± 0.01^c^	2.31 ± 0.01^d^
Ash	2.94 ± 0.03^e^	3.40 ± 0.01^d^	3.97 ± 0.04^b^	4.23 ± 0.02^a^	2.82 ± 0.01^f^	3.04 ± 0.01^d^	3.82 ± 0.02^c^	4.06 ± 0.03^b^
Soluble dietary fiber	2.46 ± 0.03^d^	2.93 ± 0.02^c^	3.28 ± 0.03^b^	3.64 ± 0.03^a^	2.33 ± 0.05^d^	2.75 ± 0.03^c^	3.16 ± 0.01^b^	3.48 ± 0.01^b^
Insoluble dietary fiber	11.88 ± 0.05^d^	12.16 ± 0.06^c^	12.77 ± 0.03^b^	13.15 ± 0.05^a^	11.60 ± 0.12^e^	11.95 ± 0.10^d^	12.69 ± 0.07^b^	12.93 ± 0.07^a^
Total dietary fiber	14.34 ± 0.13^g^	15.09 ± 0.10^e^	16.05 ± 0.17^c^	16.79 ± 0.12^a^	13.93 ± 0.10^d^	14.70 ± 0.14^f^	15.85 ± 0.12^d^	16.41 ± 0.15^b^
Amylose	21.20 ± 0.05^a^	20.93 ± 0.07^c^	20.29 ± 0.06^e^	20.03 ± 0.07^c^	21.44 ± 0.02^b^	20.81 ± 0.01^c^	20.46 ± 0.01^d^	20.33 ± 0.01^de^
Total sugar	1.15 ± 0.00^c^	1.26 ± 0.01^c^	1.37 ± 0.02^b^	1.50 ± 0.01^a^	1.26 ± 0.01^c^	1.43 ± 0.01^b^	1.59 ± 0.01^a^	1.64 ± 0.00^a^
Total starch	57.32 ± 0.11^a^	57.01 ± 0.23^b^	52.90 ± 0.21^e^	50.23 ± 0.14^f^	57.60 ± 0.24^a^	56.48 ± 0.19^c^	53.20 ± 0.20^d^	50.77 ± 0.26^f^
IVSD	69.77 ± 0.15^b^	69.20 ± 0.11^d^	66.55 ± 0.16^f^	64.17 ± 0.10^h^	70.19 ± 0.63^a^	69.62 ± 0.57^c^	67.73 ± 0.91^e^	65.08 ± 0.45^g^
RS	3.82 ± 0.03^c^	3.91 ± 0.00^b^	4.17 ± 0.03^a^	4.25 ± 0.01^a^	3.51 ± 0.01^e^	3.74 ± 0.01^d^	3.97 ± 0.01^b^	3.81 ± 0.01^c^
RDS	24.15 ± 0.07^b^	23.80 ± 0.11^d^	20.98 ± 0.15^f^	18.03 ± 0.10^g^	25.34 ± 0.11^a^	24.08 ± 0.13^c^	22.50 ± 0.19^e^	20.86 ± 0.16^f^
SDS	42.90 ± 0.12^d^	43.34 ± 0.10^c^	44.12 ± 0.15^a^	44.20 ± 0.12^a^	42.69 ± 0.20^d^	43.16 ± 0.17^d^	43.98 ± 0.22^b^	43.53 ± 0.13^c^

Mean ± standard deviation, n = 3. Values followed by the same letters in the same row are not significantly (p ≥ 0.05). IVSD = *In vitro* starch digestibility, RS = Resistant starch, RDS: rapidly digestible starch, SDS: slowly digestible starch.

### 3.2 Amylose, total sugar, and starch fractions

Amylose and total starch content significantly reduced in both varieties as germination progressed, likely due to the hydrolysis of starch to monosaccharides by enzymes such as α-amylase, activated during germination, accounting for the 30.43% and 30.16% increase in total sugar content in the brown and white fonio varieties, respectively, after 72 h of germination. Complex carbohydrates are usually broken down into simpler sugars such as maltose and maltotriose, which are used for metabolism during the germination process. The rapidly digested starch (RDS) value significantly decreased, while slowly-digestible and resistant starches increased, as the germination period increased in both varieties. The RDS was reduced by 8.02% and 7.28% while the resistant starch increased by 11.26% and 8.55%, respectively, in the brown and white varieties, after 72 h of germination. This contradicts the findings of Singh et al. [3] in their investigation into the germination of sorghum. Slowly digestible starches ensure the slow and sustained release of blood sugar, resulting in a low glycemic response necessary to control health conditions such as diabetes and obesity. Resistant starches, important in supporting a healthy gut microbiome, were significantly higher in the raw brown variety (3.82 g/100 g) than in the white variety (3.51 g/100 g) of fonio and were higher in the germinated grains of both varieties. This suggests that the brown fonio variety and germinated grains of either of the fonio varieties are potentially functional ingredients.

### 3.3 Antinutritional factors and mineral composition

Phytic acid, tannin and total saponin contents of the raw and germinated brown and white varieties of fonio are presented in Table 2. Germination time affected the antinutritional composition of the fonio grains. The antinutritional factors decreased significantly with an increase in germination time in both varieties. These antinutritional factors were higher in the white fonio than the brown variety. Phytic acid, which forms insoluble complexes with minerals elements such as Ca, Zn and Fe, making them unavailable for absorption by the body, was reduced by 73.60% and 68.16% in the brown and white varieties, respectively, after 72 h of germination compared to their respective raw samples. This reduction in phytic acid content observed during germination may be attributed partly to the activation of enzymes such as phytase during germination, which led to its breakdown [32]. Tannin, a compound which forms complexes with important nutrients such as proteins and minerals such as iron, making them unavailable for absorption, was reduced by 38.89% and 56.52% in the brown and white varieties, respectively, after 72 h germination. This reduction in antinutritional factors may have led to the significant increase in calcium, iron, magnesium, potassium, phosphorus and zinc contents which occurred over the germination period in both varieties. The calcium content increased by 14.51% and 15.63%, iron by 22.87% and 13.88%, magnesium by 25.19% and 23.19%, potassium by 19.95% and 16.57% and phosphorus by 34.86% and 22.93% in the brown and white fonio varieties, respectively, after 72 h germination. Although not investigated in this study, this level of decrease in antinutritional factors and subsequent increase in mineral content may have also led to an improvement in mineral bioaccessibility. An increase in these minerals as noted in the germinated samples is beneficial for the normal functioning of the body, ranging from building and maintaining healthy bones and teeth to keeping the muscles, heart and brain working properly. Sodium content decreased by 21.54% and 17.52% after 72 h of germination in the brown and white varieties, respectively. This reduction in sodium content is desirable, as high sodium content has been linked to adverse health conditions, including high blood pressure. The raw brown variety of fonio was higher in calcium, iron, potassium, and zinc contents and significantly lower in phosphorus and sodium content, while the magnesium content of the two varieties were not significantly different. This concurs with the findings of our ash content investigation (Table 1).

**Table 2:** Antinutritional factors, mineral composition and antioxidant activity of two varieties of native and germinated fonio flour.

Samples	Brown variety				White variety			
	Raw	24 h	48 h	72 h	Raw	24 h	48 h	72 h
Antinutritional factors								
Phytic acid (mg/100 g)	506.13 ± 1.92^b^	483.55 ± 1.67^c^	205.40 ± 1.28^f^	133.62 ± 1.21^d^	543.60 ± 2.58^a^	462.41 ± 3.11^d^	238.72 ± 2.83^e^	173.50 ± 2.06^g^
Tannin (mg CE/g)	0.18 ± 0.00^a^	0.14 ± 0.01^b^	0.12 ± 0.01^c^	0.11 ± 0.00^c^	0.23 ± 0.01^a^	0.17 ± 0.01^b^	0.12 ± 0.01^c^	0.10 ± 0.01^c^
Saponin (mg/100 g)	6.29 ± 0.01^b^	5.06 ± 0.02^d^	4.11 ± 0.01^e^	3.43 ± 0.01^g^	6.81 ± 0.03^a^	5.34 ± 0.05^c^	4.02 ± 0.07^e^	3.69 ± 0.04^f^
Mineral composition								
Calcium (mg/100 g)	32.53 ± 0.11^e^	33.90 ± 0.14^d^	36.32 ± 0.08^b^	37.25 ± 0.06^a^	31.48 ± 0.11^f^	33.63 ± 0.15^d^	35.02 ± 0.10^c^	36.40 ± 0.17^b^
Iron (mg/100 g)	3.28 ± 0.01^c^	3.46 ± 0.03^c^	3.97 ± 0.01^a^	4.03 ± 0.02^a^	3.17 ± 0.03^d^	3.25 ± 0.01^c^	3.74 ± 0.01^b^	3.61 ± 0.01^b^
Magnesium (mg/100 g)	148.41 ± 0.10^g^	158.20 ± 0.11^e^	173.53 ± 0.19^c^	185.80 ± 0.13^a^	148.63 ± 0.47^g^	157.91 ± 0.23^f^	170.33 ± 0.54^d^	183.10 ± 0.27^b^
Potassium (mg/100 g)	267.25 ± 0.93^f^	269.08 ± 1.17^e^	296.61 ± 1.02^b^	320.57 ± 1.20^a^	252.80 ± 1.13^h^	263.47 ± 1.06^g^	285.50 ± 1.19^d^	294.68 ± 1.10^c^
Phosphorus (mg/100 g)	218.47 ± 1.06^f^	226.92 ± 1.32^e^	278.30 ± 1.27^b^	294.62 ± 1.33^a^	225.44 ± 1.26^e^	236.18 ± 1.10^d^	264.61 ± 1.10^c^	277.13 ± 1.05^b^
Sodium (mg/100 g)	1.30 ± 0.00^a^	1.25 ± 0.00^a^	1.13 ± 0.01^b^	1.02 ± 0.01^c^	1.37 ± 0.01^a^	1.34 ± 0.01^a^	1.22 ± 0.01^b^	1.13 ± 0.01^b^
Zinc (mg/100 g)	2.26 ± 0.00^d^	2.41 ± 0.02^c^	2.60 ± 0.03^b^	2.88 ± 0.05^a^	2.15 ± 0.01^d^	2.29 ± 0.01^d^	2.41 ± 0.01^c^	2.65 ± 0.01^b^
Antioxidant activity								
TPC (mg/GAE/100g)	19.10 ± 0.15^g^	28.45 ± 0.12^e^	63.90 ± 0.17^c^	75.88 ± 0.14^a^	18.43 ± 0.11^h^	25.77 ± 0.19^f^	54.68 ± 0.26^d^	69.90 ± 0.31^b^
DPPH (μmol TE/100g)	53.62 ± 0.28^g^	72.77 ± 0.33^e^	86.23 ± 0.14^c^	95.57 ± 0.19^a^	51.86 ± 0.16^h^	68.25 ± 0.12^f^	83.19 ± 0.14^d^	92.53 ± 0.10^b^
ORAC (μM TE/100 g)	3113.60 ± 6.07^g^	3269.16 ± 10^e^	3581.83 ± 8.05^c^	3704.10 ± 7.42^a^	3062.55 ± 14.28^h^	3310.47 ± 13.06^f^	3490.66 ± 12.19^d^	3678.20 ± 13.41^b^

Mean ± standard deviation, n = 3. Values followed by the same letters in the same row are not significantly (p ≥ 0.05). TPC = Total phenolic content; DPPH = 1,1-diphenyl-2-picryl-hydrazil radical scavenging activity; ORAC = Oxygen radical absorbance capacity.

### 3.4 Total phenolic content and antioxidant activity

Phenolic compounds do not possess nutritional properties, but instead scavenge free radicals in the human system, which promotes health. The total phenolic content significantly (p ≤ 0.05) increased by 297.28% and 279.27% in the brown and white varieties, respectively, compared to their raw samples; this is likely due to the breakdown of the cell walls during germination, leading to the liberation of bound phenolic compounds [20]. A similar observation has been noted in sorghum following germination [3]. Antioxidant activities usually result from the presence of phenolic compounds and vitamins. The increase in total phenolic content could have been the reason for the significant increase observed in DPPH radical scavenging activity and oxygen radical absorbance capacity of the fonio samples with the increase in germination period, compared to their raw samples. The DPPH values significantly increased by 78.24% and 78.42%, while the ORAC increased by 18.97% and 20.10% in the brown and white fonio varieties, respectively, after 72 h of germination, compared to their raw samples. Changes in the antioxidant activities could have also been due to the hydrolysis of larger chemical structures and biotransformation, which might have occurred during germination, generating new bioactive compounds which contributed to increased antioxidant activities. The improvement of antioxidant activity of the two fonio varieties after germination suggests potential health-promoting properties.

### 3.5 Amino acid composition

The amino acid composition of the raw and germinated brown and white fonio varieties is shown in Table 3. Among the essential amino acids, leucine content was the highest, 0.63−0.82 g/100 g (brown variety) and 0.61−0.78 g/100 g (white variety), while histidine was the lowest, 0.22−0.29 g/100g and 0.23−0.28 g/100g, in the brown and white varieties, respectively. Glutamic acid was the most abundant of the non-essential amino acids, 1.36−1.78 g/100 g and 1.34−1.49 g/100g, while cysteine was the least abundant, 0.13−0.21 g/100 g and 0.15−0.17 g/100 g, in the brown and white varieties, respectively. There was no significant difference between the histidine, cysteine, glycine and serine contents of the raw and germinated fonio samples in both varieties. However, the other amino acids (isoleucine, leucine, lysine, methionine, phenylalanine, threonine, valine, alanine, arginine, aspartic acid and tyrosine) were significantly higher in the germinated samples than in the raw samples. The contents did not vary significantly with increased germination time except for glutamic acid, which significantly increased with an increase in germination time. The improvement in amino acids observed may be linked to changes that occurred during germination, including the reduction in antinutritional factors (as observed in Table 2) which could have led to the release of certain previously bound amino acids [20].

**Table 3:** Amino acid composition (g/100 g) properties of two varieties of native and germinated fonio flour.

Samples	Brown variety				White variety			
	Raw	24 h	48 h	72 h	Raw	24 h	48 h	72 h
Histidine	0.22 ± 0.01^a^	0.26 ± 0.01^a^	0.28 ± 0.01^a^	0.29 ± 0.01^a^	0.23 ± 0.00^a^	0.25 ± 0.01^a^	0.27 ± 0.01^a^	0.28 ± 0.01^a^
Isoleucine	0.35 ± 0.01^b^	0.51 ± 0.02^a^	0.53 ± 0.01^a^	0.52 ± 0.00^a^	0.36 ± 0.01^b^	0.49 ± 0.02^a^	0.51 ± 0.01^a^	0.50 ± 0.00^a^
Leucine	0.63 ± 0.00^b^	0.79 ± 0.01^a^	0.81 ± 0.01^a^	0.82 ± 0.01^a^	0.61 ± 0.01^b^	0.77 ± 0.01^a^	0.79 ± 0.01^a^	0.78 ± 0.02^a^
Lysine	0.25 ± 0.01^b^	0.35 ± 0.01^a^	0.38 ± 0.02^a^	0.38 ± 0.01^a^	0.24 ± 0.01^b^	0.33 ± 0.01^a^	0.36 ± 0.00^a^	0.37 ± 0.01^a^
Methionine	0.22 ± 0.01^b^	0.29 ± 0.01^a^	0.33 ± 0.01^a^	0.35 ± 0.01^a^	0.20 ± 0.01^b^	0.28 ± 0.01^a^	0.33 ± 0.02^a^	0.34 ± 0.01^a^
Phenylalanine	0.30 ± 0.01^b^	0.39 ± 0.01^a^	0.46 ± 0.01^a^	0.47 ± 0.01^a^	0.31 ± 0.01^b^	0.38 ± 0.02^a^	0.45 ± 0.01^a^	0.46 ± 0.02^a^
Threonine	0.24 ± 0.00^b^	0.38 ± 0.01^a^	0.39 ± 0.01^a^	0.41 ± 0.02^a^	0.23 ± 0.01^b^	0.36 ± 0.01^a^	0.38 ± 0.01^a^	0.39 ± 0.01^a^
Valine	0.43 ± 0.02^b^	0.55 ± 0.03^a^	0.54 ± 0.01^a^	0.58 ± 0.02^a^	0.41 ± 0.01^b^	0.54 ± 0.01^a^	0.55 ± 0.01^a^	0.57 ± 0.01^a^
Alanine	0.53 ± 0.02^b^	0.67 ± 0.03^a^	0.69 ± 01^a^	0.74 ± 0.01^a^	0.49 ± 0.01^b^	0.65 ± 0.01^a^	0.68 ± 0.01^a^	0.71 ± 0.02^a^
Arginine	0.36 ± 0.01^b^	0.45 ± 0.01^a^	0.58 ± 0.01^a^	0.60 ± 0.00^a^	0.35 ± 0.01^b^	0.48 ± 0.02^a^	0.55 ± 0.02^a^	0.57 ± 0.01^a^
Aspartic acid	0.53 ± 0.01^b^	0.64 ± 0.01^a^	0.67 ± 0.02^a^	0.68 ± 0.01^a^	0.50 ± 0.02^b^	0.61 ± 0.01^a^	0.64 ± 0.01^a^	0.60 ± 0.00^a^
Cysteine	0.13 ± 0.01^a^	0.15 ± 0.00^a^	0.19 ± 0.01^a^	0.21 ± 0.02^a^	0.15 ± 0.02^a^	0.16 ± 0.01^a^	0.17 ± 0.01^a^	0.17 ± 0.01^a^
Glutamic acid	1.36 ± 0.03^d^	1.47 ± 0.01^c^	1.63 ± 0.01^b^	1.78 ± 0.02^a^	1.34 ± 0.01^b^	1.41 ± 0.01^a^	1.45 ± 0.01^a^	1.49 ± 0.02^a^
Glycine	0.27 ± 0.01^a^	0.29 ± 0.01^a^	0.28 ± 0.00^a^	0.30 ± 0.01^a^	0.26 ± 0.00^a^	0.28 ± 0.01^a^	0.29 ± 0.01^a^	0.28 ± 0.01^a^
Proline	0.34 ± 0.03^c^	0.49 ± 0.01^b^	0.63 ± 0.01^a^	0.68 ± 0.01^a^	0.39 ± 0.01^c^	0.47 ± 0.00^b^	0.60 ± 0.01^a^	0.66 ± 0.02^a^
Serine	0.38 ± 0.01^a^	0.46 ± 0.01^a^	0.48 ± 0.02^a^	0.51 ± 0.02^a^	0.41 ± 0.02^a^	0.45 ± 0.01^a^	0.47 ± 0.02^a^	0.48 ± 0.01^a^
Tyrosine	0.21 ± 0.01^b^	0.30 ± 0.01^a^	0.32 ± 0.01^b^	0.45 ± 0.01^a^	0.23 ± 0.01^b^	0.36 ± 0.01^a^	0.40 ± 0.01^a^	0.45 ± 0.01^a^
IVPD (%)	75.49 ± 0.24^d^	76.51 ± 0.16^c^	78.80 ± 0.22^b^	79.67 ± 0.19^a^	74.16 ± 0.37^d^	75.80 ± 0.24^c^	77.54 ± 0.39^b^	79.22 ± 0.20^a^

Mean ± standard deviation, n = 3. Values followed by the same letters in the same row are not significantly (p ≥ 0.05). IVPD = In vitro protein digestibility.

The *in vitro* protein digestibility significantly increased by 5.54% and 6.82% in the brown and white variety, respectively, after 72 h of germination, and could likely be ascribed to the increased activity of protease enzymes during germination, causing structural changes in the storage protein, thereby making it more susceptible to hydrolysis, thus decreasing the molecular size of proteins and increasing the *in vitro* protein digestibility. A similar increase in protein digestibility after germination has been reported in other cereals, including sorghum [3], amaranth and quinoa, amaranth and finger millet [4] and brown finger millet [20].

### 3.6 Functional, pasting, and thermal properties

The functional, pasting, and thermal properties of raw and germinated fonio are shown in Table 4. Bulk density decreased by 46.99% and 46.75% in the brown and white varieties, respectively, as germination progressed, probably due to the modification of macro constituents such as dietary fiber, starch, and proteins. The water absorption capacity (WAC), water solubility index (WSI) and oil absorption capacity (OAC) increased significantly with an increase in germination time in both varieties. The WSI increased by 465.45% and 465.36% in the brown and white varieties after 72 h of germination, partly due to the breakdown of polysaccharides into monosaccharides, which are more soluble. A similar increase in WSI has been reported in amaranth following 24 h of germination [2]. The WAC increased by 28.83% and 43.49%, respectively, in the brown and white varieties after 72 h germination, which was attributed to the increased activity of amylolytic enzymes which break down starch [33]. An increase in the WAC of flour could be beneficial in bread making, because high water absorption has a positive impact on dough capacity and bread volume [27]. The increase in OAC, by 40.59% and 40.78% following germination in the brown and white fonio varieties, respectively, may be due to the increase in hydrophobic proteins and fats which bond with oil, present in the germinated flours samples [16,34]. This could impact flavor during product development [16].

**Table 4:** Functional, pasting and thermal properties of two varieties of native and germinated fonio flour.

Samples	Brown variety				White variety			
	Raw	24 h	48 h	72 h	Raw	24 h	48 h	72 h
Functional properties								
Bulk density (g/cm3)	0.83 ± 0.01^a^	0.73 ± 0.02^b^	0.58 ± 0.00^c^	0.44 ± 0.02^d^	0.77 ± 0.03^a^	0.70 ± 0.01^b^	0.56 ± 0.01^c^	0.41 ± 0.01^d^
WAC (g/g)	2.79 ± 0.02^d^	3.06 ± 0.01^c^	3.47 ± 0.02^b^	3.92 ± 0.01^a^	2.92 ± 0.01^d^	3.15 ± 0.03^c^	3.72 ± 0.01^b^	4.19 ± 0.02^a^
WSI (g/g)	9.03 ± 0.07^d^	27.10 ± 0.05^c^	35.92 ± 0.04^b^	51.06 ± 0.07^a^	9.15 ± 0.11^d^	27.42 ± 0.18^c^	36.06 ± 0.09^b^	51.73 ± 0.15^a^
OAC (g/g)	1.01 ± 0.01^d^	1.20 ± 0.00^c^	1.30 ± 0.01^b^	1.42 ± 0.00^a^	1.03 ± 0.01^d^	1.20 ± 0.01^c^	1.33 ± 0.01^b^	1.45 ± 0.01^a^
Pasting properties								
Peak viscosity (cP)	2165 ± 1.21^b^	1691 ± 1.03^d^	1153 ± 1.24^f^	575 ± 1.06^h^	2260 ± 1.34^a^	1742 ± 1.17^c^	1225 ± 1.14^e^	618 ± 1.19^g^
Trough viscosity (cP)	1745 ± 1.33^b^	1309 ± 1.25^d^	882 ± 1.20^f^	471 ± 0.94^h^	1802 ± 1.15^a^	1340 ± 1.23^c^	932 ± 1.31^e^	506 ± 1.17^g^
Break down (cP)	440 ± 1.10^b^	382 ± 1.13^d^	271 ± 0.97^f^	104 ± 0.82^h^	458 ± 1.30^a^	402 ± 0.95^c^	293 ± 1.27^e^	112 ± 1.03^g^
Final viscosity (cP)	2389 ± 1.24^b^	1746 ± 1.21^d^	1250 ± 1.33^f^	722 ± 1.20^h^	2433 ± 1.86^a^	1854 ± 1.13^c^	1340 ± 1.42^e^	779 ± 1.65^g^
Setback viscosity (cP)	664 ± 0.75^a^	437 ± 0.81^d^	368 ± 0.91^f^	301 ± 0.53^g^	631 ± 1.06^b^	514 ± 1.17^c^	408 ± 1.21^e^	273 ± 1.32^h^
Pasting temperature (oC)	77.42 ± 0.08^e^	78.05 ± 0.05^d^	78.39 ± 0.03^c^	78.70 ± 0.16^b^	77.81 ± 0.12^d^	78.30 ± 0.17^c^	78.83 ± 0.10^b^	79.10 ± 0.16^a^
Peak time (Min)	6.39 ± 0.03^a^	6.20 ± 0.01^ab^	6.01 ± 0.01^c^	5.68 ± 0.01^d^	6.46 ± 0.07^a^	6.29 ± 0.03^b^	6.05 ± 0.03^c^	5.73 ± 0.03^d^
Thermal properties								
Onset temperature (oC)	65.73 ± 0.17^h^	69.50 ± 0.10^f^	71.36 ± 0.14^d^	75.60 ± 0.05^b^	66.35 ± 0.13^g^	70.81 ± 0.14^e^	72.17 ± 0.11^c^	77.51 ± 0.08^a^
Peak temperature (oC)	66.51 ± 0.22^h^	70.85 ± 0.12^f^	72.44 ± 0.10^d^	78.19 ± 0.14^b^	67.92 ± 0.15^g^	71.46 ± 0.10^e^	73.30 ± 0.19^c^	79.69 ± 0.12^a^
Conclusion temp. (oC)	67.60 ± 0.20^h^	73.49 ± 0.17^f^	74.61 ± 0.19^d^	80.43 ± 0.10^b^	68.77 ± 0.20^g^	74.02 ± 0.15^e^	75.46 ± 0.23^c^	81.25 ± 0.18^a^
Enthalpy change (J/g)	8.83 ± 0.11^f^	10.26 ± 0.14^e^	11.73 ± 0.10^d^	12.31 ± 0.15^c^	9.12 ± 0.07^d^	11.58 ± 0.04^d^	12.83 ± 0.09^b^	13.10 ± 0.03^a^

Mean ± standard deviation, n = 3. Values followed by the same letters in the same row are not significantly (p ≥ 0.05). WAC = Water absorption capacity, WSI = Water solubility index, OAC= Oil absorption capacity.

The peak viscosity, trough, breakdown, setback, and final viscosities all reduced (p < 0.05) with an increase in germination time in both varieties (Table 3), partly due to increased amylase activity, which hydrolyses starch to shorter chain dextrins during germination. This leads to a reduction in total starch, which plays a significant role in the pasting characteristics of flour [35]. A similar reduction in pasting viscosities was reported by Cornejo et al. [2] in two amaranth species, *Amaranthus quitensis* and *Amaranthus caudatus*, after 24 h of germination*.* The decreased pasting viscosities in the white and brown fonio varieties could be of relevance in weaning food formulation and baking. There is scientific evidence that flour with low paste viscosities could be advantageous in wheat-based composite flours for bread production [36]. Peak viscosity reflects the swelling power of starch particles before its breakdown and plays an important role in the textural quality of final products. Breakdown viscosity shows the thermal stability of starch granules. The higher the breakdown viscosity, the lower the resistance of the starch to shear thinning during cooking, and the easier the starch slurry decomposes. The white variety of fonio had a higher breakdown viscosity than the brown variety. The reduction (p ≤ 0.05) in breakdown viscosity as germination progressed suggests that starches from germinated fonio have higher hot-paste stability than the native starches. The raw white variety had a higher final viscosity (2433 cP) than the raw brown variety (2389 cP). There was a significant increase in the onset (15.02% and 16.82%), peak (17.56% and 17.33) and conclusion (18.99% and 18.15%) temperatures with the increase in germination time in both the brown and white varieties. This may be due to the breakdown of starch by α- and β-amylases and α-glucosidase leading to the production of low molecular weight dextrins with poor swelling properties, resulting in a higher gelatinization temperature [37]. The enthalpy change increased significantly with an increase in germination time in both varieties.

### 3.7 Principal component analysis (PCA) of chemical, nutritional and functional properties data

**Fig. 1:**
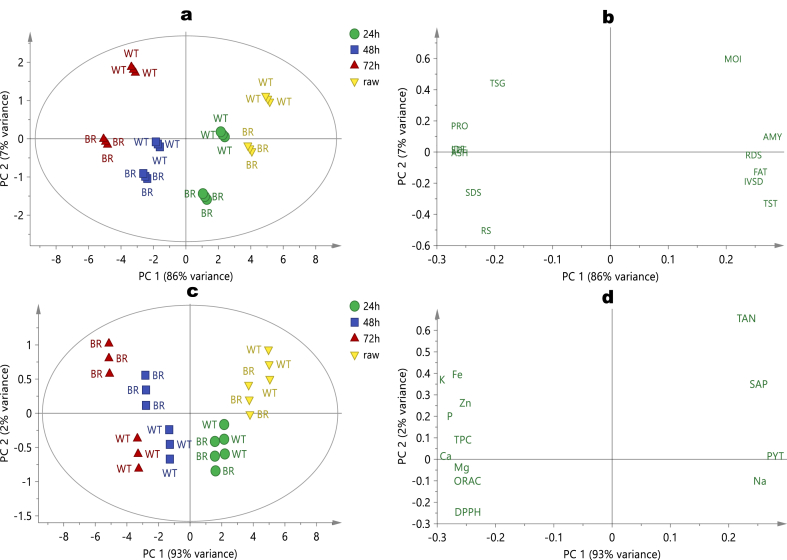
Multivariate PCA model score (a) and loading (b) plots of chemical composition data; score (c) and loading (d) plots of antinutritional factors, mineral composition, and antioxidant activity data of ungerminated (Raw) and germinated (24 h; 48 h; and 72 h) fonio millet flour obtained from two varieties (white: WT; and brown: BR).

Principal component analysis (PCA) models were created to provide a comprehensive picture of the multivariate differences in fonio regarding variety and germination time. Four separate PCA data matrices comprising of chemical compositions (24 samples and 14 variables), antinutritional factors, mineral compositions, and antioxidant activity (24 samples and 14 variables), amino acid composition (24 samples and 18 variables) and finally, functional, pasting, and thermal properties (24 samples and 15 variables) were built. Score and loading plots were only presented for the top two principal components (PC), which represented more than 90% of the data variability in all the models. In the case of chemical composition, the two PC explained 93% of the data variance, with a coefficient of cross-validation (Q^2^) of 0.96. The patterns of samples distribution (Fig. 1a) and variable projections (Fig. 1b) in the score and loading plots indicated a recognizable pattern which can be summarized into the four ellipse quadrants. Raw and 24 h germinated brown and white fonio occupy the right positive plane of PC 1 and PC 2. According to the loading plots, the proximity of their clusters shows similarities in their IVSD, RDS, fat, starch, amylose, and moisture contents. These overlaps are more obvious in the raw brown fonio and 24 h germinated white fonio. However, the raw white fonio is distinguished by its unusually high moisture content.

**Fig. 2:**
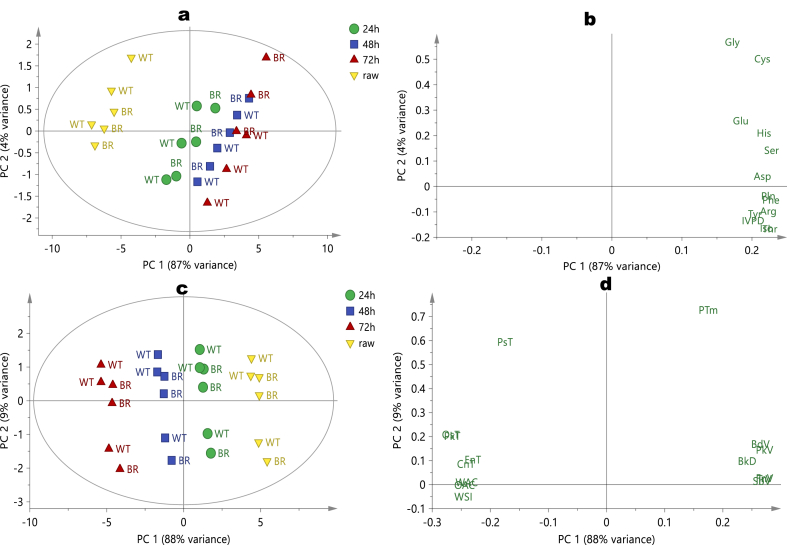
Multivariate PCA model’s score (a) and loading (b) plots of chemical composition data; score (c) and loading (d) plots of antinutritional factors, mineral composition, and antioxidant activity data of ungerminated (Raw) and germinated (24 h; 48 h; and 72 h) fonio millet flour obtained from two varieties (white: WT; and brown: BR).

On the other hand, the negative plane of PC 1 and PC 2 contained the 48 and 72 h germinated white and brown fonio. Variables such as SDS and RS were significantly high, especially in 48 h germinated brown fonio. Interestingly, at 72 h germination, the samples exhibited high dietary fiber (total, soluble and insoluble) and ash contents, particularly among brown fonio. During germination, inherent hydrolysable macromolecules are predisposed to hydrolysis and eventual quantitative reductions [1,20]. Therefore, non-hydrolysable components such as fibre, minerals, and hydrolytic by-products such as sugar, may become more pronounced, as shown in the loading. Fig. 1c and d were generated from PCA (2 PCs explained 95% variance, Q^2^ = 0.92) models of the antinutrient, mineral and antioxidant capacity data of millets. Raw brown and white millets clustered together on the upper right quadrant of PC 1 with antinutrients such as tannin, saponin and phytate are responsible for this separation. As recently reported [20], germination is an enzyme-mediated process leading to hydrolysis and the reduction of antinutrients. In the orthogonal direction, germination between 48 and 72 h caused a significant increase in antioxidant capacity (ORAC and DPPH) and the magnesium content of white fonio, while the same germination conditions resulted in high TPC, Ca, K, Fe, and P contents of brown millet. High Na is the most defining properties of 24 h germinated millet regardless of variety. Fig. 2a and b shows score and loading plots of PCA built on an amino acids profile of the samples. As with the previous matrices, the model parameters include 99% explained variance by 2 PCs and Q^2^ of 0.97. There were two somewhat distinct clusters in the score plot (raw millets and others). None of the amino acids were high enough to define raw fonio. As shown in the score, the influence of variety on amino acid distribution was low. However, as germination time extends, individual amino acids and *in vitro* protein digestibility increased, as shown in the vertical orientation of 24 h, 48 h and 72 h germinated samples from left to right on the control ellipse. Other studies have also reported the positive influence of germination on protein and amino acids profiles in cereals and pseudo-cereals [38,39].

In the PCA score and loading plots for functional and thermal properties (Fig. 2c and d), the sample cluster was based mainly on germination time, rather than on variety, with the positive end of PC1 and PC 2 occupied by raw and 24 h germinated fonio of both varieties. These samples are best represented by their high peak, breakdown, set back, and trough viscosities, pasting time and bulk density. Thus, as expected of a native starchy matrix, the flours from raw fonio required a greater time and viscosity to reach maximum starch gelatinization compared to the enzymatically hydrolyzed matrix in the germinated samples. Therefore, as germination time increased, the viscosities decreased, which concurs with the observations of Wang et al. [40] regarding rice. However, regardless of the variety, fonio germinated for between 48 and 72 h were characterized by high values of pasting, onset, conclusion and peak temperatures, enthalpy change, water and oil absorption capabilities, as shown in the left quadrant of the score and loading plots. The temperature at which the viscosity begins to rise during the heating process is referred to as the pasting temperature, and starchy matrices with a high pasting temperature have a stronger resilience to swelling and rupture [41]. Therefore, in addition to having a high WAC and OAC, the longer the germination period, the less suitable it is for the production of thinner gruel for infant formulas.

### 3.8 Regression and VIP modeling of *in vitro* starch and protein digestibility

**Fig. 3:**
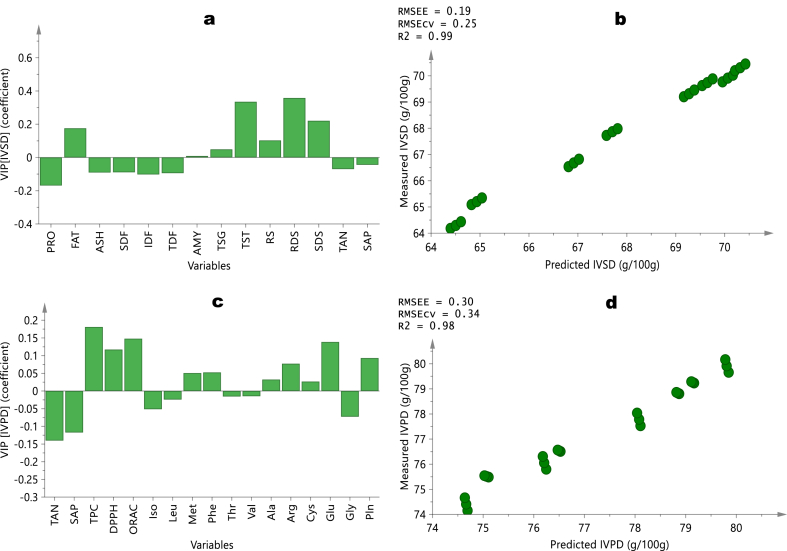
PLS prediction results of *in vitro* starch (IVSD) and protein (IVPD) digestibility of Fonio Millet Flour. The loading weights of chemical variables of the regression model (a) and scatter plot of predicted vs measured IVSD (b); the loading weights of amino acids and antioxidant variables of the regression model (c) and scatter plot of predicted vs measured IVPD (d); RMSEE: root mean square error of estimation; RMSE_CV_: root mean square of the cross validation.

The contributions of other quality variables to *in vitro* protein and starch digestibility in fonio millets were calculated using variable important projection (VIP) algorithms and partial least squares regression (PLSR) models. A sub-set of the most informative variables based on the VIP weight and chemically related parameters were selected to avoid over-fitting and to ensure the heteroscedasticity of the models. A VIP weight is considered significant if it is above one (1), while those below 0.5 are deemed to be less significant [29]. The magnitude and direction of projection of a VIP coefficient determines the influence of the independent variable (predictor) and the dependent variable (predicted). Fig. 3 shows the VIP coefficients of the dependent variables, in this case, IVPD and IVSD, against the selected (VIP weight >0.5) independent variables and PLSR line graph. In the case of IVSD (Fig. 3a−b), the primary deciding variables impacting starch digestibility were rapidly digestible starch (RDS), total starch (TST), and slowly digested starch (SDS). The greater the amount of RDS in fonio millet, the higher the digestibility. In addition, the model's robustness as highlighted by a strong regression coefficient of determination (R^2^ = 0.99) and a low root mean square error of prediction and cross-validation (RMSEE = 0.19 and RMSEcv = 0.25).

Interestingly, all types of dietary fibre (soluble, insoluble, and total: SDF, IDF, TDF), together with protein, ash, tannin and saponin, decreased starch digestion to a minor degree, as seen by their negative contributions. Protein digestibility is most influenced by total phenol content (TPC), DPPH and ORAC antioxidant activities, tannin, glutamic acid, and saponin and the model’s performance parameters were satisfactory (R^2^ = 0.98; RMSEE = 0.30 and RMSEcv = 0.34), as shown in Fig. 3c and d. The most influential positive predictors were the phenolic compounds and antioxidants, and tannin and saponin significantly affected IVPD negatively. Tannins have often been reported to reduce protein digestibility and essential amino acid levels by forming temporary and permanent tannin-protein interactions via their side chains, while saponins also hinder protease enzymes, causing indigestion [42]. The negative impacts of polyphenols on protein digestibility stem from their binding to both digestive enzymes and protein substrates [43]. Therefore, the high quantifiable free polyphenol in fonio with high IVPD is an indication of low protein-phenol interaction.

## 4 Conclusion

The nutritional and physicochemical changes in white and brown varieties of fonio were evaluated. The results demonstrated that the nutrient composition, total phenolics, antioxidant activities, resistant starch, protein digestibility of fonio improved with an increase in germination time, with a 72 h germination period providing the highest value in both brown and white fonio varieties, while phytic acid, tannin and total saponin content decreased. The functional, pasting and thermal properties were slightly modified after germination. The low viscosity, high water and oil absorption capacity of the 72 h germinated flours from both varieties could be beneficial when pairing with wheat flour in bread production. In addition, the low viscosity of the 72-h germinated flour could be useful in weaning food formulations, where reduced paste viscosity plays a key role in the final product quality. The principal component analysis revealed germination time to be the key determinant in the physicochemical, nutritional, and functional characteristics of fonio rather than variety, with the raw and the 24 h germinated fonio grains having similar attributes, while increased germination time modified the techno-functional parameters evaluated. Rapidly digestible and total starch content exerted the most positive influence on starch digestibility, while polyphenol and antioxidant activities contributed to the protein digestibility of fonio. Further research is required to profile the structural and physicochemical properties of the germinated fonio starch from both varieties in order to elucidate these changes.

## Author contribution statement

Stella Oyom Bassey, Vanessa Chinelo Ezeocha, Olajide Emmanuel Adedeji, Olusola Samuel Jolayemi, Uzoamaka Christa Alozie-Uwa, Irene Eneyi Adie, Salvation Isang Ofem: Performed the experiments; Analyzed and interpreted the data; Wrote the paper.

Chiemela Enyinnaya Chinma: Conceived and designed the experiments; Analyzed and interpreted the data; Contributed reagents, materials, analysis tools or data; Wrote the paper.

Janet Adeyinka Adebo, Oluwafemi Ayodeji Adebo: Contributed reagents, materials, analysis tools or data; Wrote the paper.

## Data availability statement

Data will be made available on request.

## Declaration of competing interest

The authors declare that they have no known competing financial interests or personal relationships that could have appeared to influence the work reported in this paper.
